# Comparative Evaluation of Surface Roughness of Different Rotary Nickel-Titanium (NiTi) Files After Autoclaving: An Atomic Force Microscopic Study

**DOI:** 10.7759/cureus.66054

**Published:** 2024-08-03

**Authors:** Angela Alex, Ranjith Kumar Sivarajan, Vijay Venkatesh

**Affiliations:** 1 Department of Conservative Dentistry and Endodontics, SRM Kattankulathur Dental College and Hospital, Chennai, IND

**Keywords:** atomic force microscope, surface roughness, autoclaving, curved canals, waveone gold, hyflex edm

## Abstract

Introduction

Canal preparation is a critical step in endodontic therapy. Introducing nickel-titanium (NiTi) rotary instruments has significantly reduced the likelihood of errors in curved canals. However, due to their price, these instruments are often reused following autoclaving. The aim of this study is to compare and evaluate the surface characteristics of two designs of rotary NiTi files used in curved canals and subjected to multiple autoclaving cycles, utilizing an atomic force microscope for detailed analysis.

Methods

A total sample size of 24 files was taken, 12 files of Hyflex EDM (Coltene/Whaledent, Germany) files and WaveOne Gold (Dentsply Sirona, USA) files were then divided into four groups (n=6) as follows: Group I: Hyflex EDM control group; Group II: WaveOne Gold control group; Group III: Hyflex EDM experimental group; Group IV: WaveOne Gold experimental group.

Sterilization using an autoclave was performed thrice for Groups I and II files. The files in Groups III and IV were used in simulated curved canals three times and autoclaved after each use. Atomic force microscopy was used to assess the surface roughness of the files after the first and third autoclave cycles.

Results

The results showed that, without statistical significance, Hyflex EDM exhibited the highest surface roughness after the first usage among the two file systems.

Conclusion

It can be concluded that both Hyflex EDM and WaveOne Gold files produced similar levels of surface changes when subjected to multiple usage and autoclaving cycles.

## Introduction

Endodontic therapy aims to preserve patients' natural teeth by treating both necrotic and viable dental pulps [1]. This process involves multiple stages, each contributing to the overall success of the treatment. One of the most crucial stages in every root canal treatment is canal preparation, which ensures the removal of infected tissue and the shaping of the canal for subsequent filling [2].

For root canal preparation, the advent of rotary nickel-titanium (NiTi) instruments has significantly improved the procedure's efficiency and safety. These instruments have demonstrated a decreased chance of procedural errors such as ledging, zipping, stripping, or transporting, which are common challenges when preparing curved canals [3]. The flexibility and resilience of NiTi files allow for better navigation and cleaning of complex canal anatomies compared to traditional stainless-steel files.

However, the use of NiTi files in curved canals presents its own set of challenges. The increased contact area between the instrument’s cutting edge and the canal wall in curved canals leads to greater cyclic fatigue [4]. Cyclic fatigue occurs due to repeated stress on the instrument as it navigates through the curved paths, which can ultimately cause the file to fracture if not managed properly. This increased cyclic fatigue is a critical factor because it directly affects the longevity and performance of the instrument.

The cyclic fatigue of an instrument increases with the degree of canal curvature, which subsequently reduces the instrument's expected lifespan [5]. The deterioration of rotary NiTi instruments has been examined in numerous studies. For instance, a study by Uslu et al. evaluated the surface topographies of two file systems, revealing that following the preparation of highly dilacerated canals, the files exhibited increased surface roughness values. This increase in surface roughness can affect the mechanical characteristics of rotary NiTi files, potentially leading to unintended fractures during root canal treatment [6].

Due to their high cost, NiTi rotary files are often reused following autoclaving. Sterilization using autoclaves can significantly reduce cross-infections during root canal therapy, as stated in the literature. A study by Valois et al. utilized an atomic force microscope to assess the surface of rotary NiTi instruments after different cycles of autoclaving. Results showed that rotary NiTi files when subjected to multiple cycles of autoclaving, increase the depth of irregularities on the surface of the files [7].

In recent years, advancements in endodontic instrument design and mechanical characteristics have significantly improved the safety and efficacy of NiTi files. Numerous innovations, such as new production techniques, have been employed to enhance the microstructure of NiTi files.

One such advancement is the Hyflex EDM (Coltene/Whaledent,Germany), a single file system produced using controlled memory alloy via electric discharge machining technology [6]. This process results in a file with superior flexibility and resistance to cyclic fatigue, making it particularly effective for navigating complex root canal anatomies.

Another example is Dentsply Maillefer's WaveOne (Dentsply Sirona, USA), a single file system that undergoes a gold heat treatment process during manufacturing. This process involves heating and subsequent cooling of the file, in contrast to the pre-manufacturing heat treatment used in M-Wire technology. This novel heat treatment enhances the flexibility of files, which is crucial for maintaining the file’s integrity in curved canals [8].

For the surface characterization of rotary NiTi files, previous studies have often employed scanning electron microscopy (SEM). However, SEM does not provide a three-dimensional image of the surface characteristics. In contrast, atomic force microscopy (AFM) has been established for use in material analysis that offers detailed topographical information. The flexible cantilever of AFM is attached to a small tip which probes the sample’s surface and identifies forces at the sample-tip interactions. This technique yields both quantitative as well as qualitative information on the topography of the sample studied, providing a comprehensive three-dimensional view [9].

Hence, the present study aimed to compare and evaluate the surface characteristics of two manufacturing designs of NiTi rotary files used in curved canals and subjected to multiple autoclaving cycles using an atomic force microscope. This evaluation will help in understanding the effect of repeated use and sterilization on the integrity and performance of these endodontic instruments.

Null hypothesis

1. There would be no difference in surface properties between the two file systems after multiple uses and autoclaving cycles.

2. The manufacturing process and the motion of the file will not have an effect on the surface properties after multiple usage and autoclaving cycles.

## Materials and methods

The present experimental study was approved by the Institutional Research Ethics Committee at the SRM Medical College Hospital and Research Center (ethical committee approval no. SRMIEC-ST0723-770).

Sample size calculation

Sample size determination was done using the Epitools calculator (Epitools, USA) with a 95% confidence level, a significance level of p≤0.05, and a desired power of 90%. The sample size calculated for one group was four and was then approximated and adjusted to six, which resulted in a total sample size of 24. Twelve files of Hyflex EDM and 12 files of WaveOne Gold were selected to be distributed equally among the control and experimental groups.

The inclusion criteria encompassed simulated canals with a curvature of 50 degrees, determined using Schneider’s method, and single file systems of Hyflex EDM and WaveOne Gold. The exclusion criteria included NiTi files with visible defects and simulated canals with defects.

Acrylic resin blocks containing simulated canals with a curvature of 50 degrees were distributed equally for usage with Hyflex EDM experimental files and WaveOne Gold experimental files. To establish the real working length (RWL), a #10 file was placed into the simulated canal until the file's tip matched the canal's end, then 1 mm was subtracted [10]. The working length was determined to be 16 mm. Following this, the canals were manually instrumented using #10 and #15 K files up to the RWL, with irrigation using saline between subsequent instrumentations.

Pre-operative assessment

A pre-operative scan was performed for six files from each of the four groups using an Anton Paar Step 700 atomic force microscope (Anton Paar, USA). The contact operation mode was employed, utilizing AFM probes with a curvature radius of 10nm mounted on a cantilever. The files were scanned at a point 3mm from the tip of the file with a spring constant of 20nN. The 3D images were analyzed using Nanosurf 3000 software (Nanosurf AG, Switzerland) to obtain the surface roughness and line roughness values.

Post-operative assessment after first cycle

Six Hyflex EDM files and six WaveOne Gold files were packed into sterile pouches and subjected to an autoclave cycle for 15 minutes at 121°C and 15 psi of pressure. The files were allowed to cool and dry out. The file samples were subjected to AFM imaging and processed using the Nanosurf 3000 software.

Post-operative assessment after third cycle

Following the first autoclave cycle and AFM scan, the same six Hyflex EDM files and six WaveOne Gold files were again packed into sterile pouches and subjected to two additional autoclave cycles. After these cycles, the file samples were subjected to AFM imaging and processed using the Nanosurf 3000 software.

Post-operative assessment after first usage

Six new Hyflex EDM 25/~ files were mounted on an endomotor (X-Smart Plus, Dentsply Sirona, USA) and six new WaveOne Gold 25/0.07 files were also mounted on an endomotor to be used in a reciprocating motion. The files were used in six different simulated canals. Following this, they were packed into sterile pouches and subjected to an autoclave cycle. AFM images of the file samples were then obtained and processed using the Nanosurf 3000 software.

Post-operative assessment after third usage

After the first usage and first autoclave cycle, the same six Hyflex EDM 25/~ files and six WaveOne Gold 25/0.07 files were mounted on an endomotor. The files were used in simulated canals and then subjected to an autoclave cycle. The same files were subsequently used again for a third time in simulated canals and subjected to another autoclave cycle. Following this, the file samples were subjected to AFM imaging and processed with Nanosurf 3000 software.

## Results

The study aimed to compare the average surface roughness (Ra) and root mean square roughness (Rq) among four different groups and to assess the changes in these parameters within each group across three time points: pre-operation (pre-op), after the first autoclave, and after the third autoclave. Statistical analyses were conducted to determine the significance of the observed differences.

Intergroup comparison

**Figure 1:**
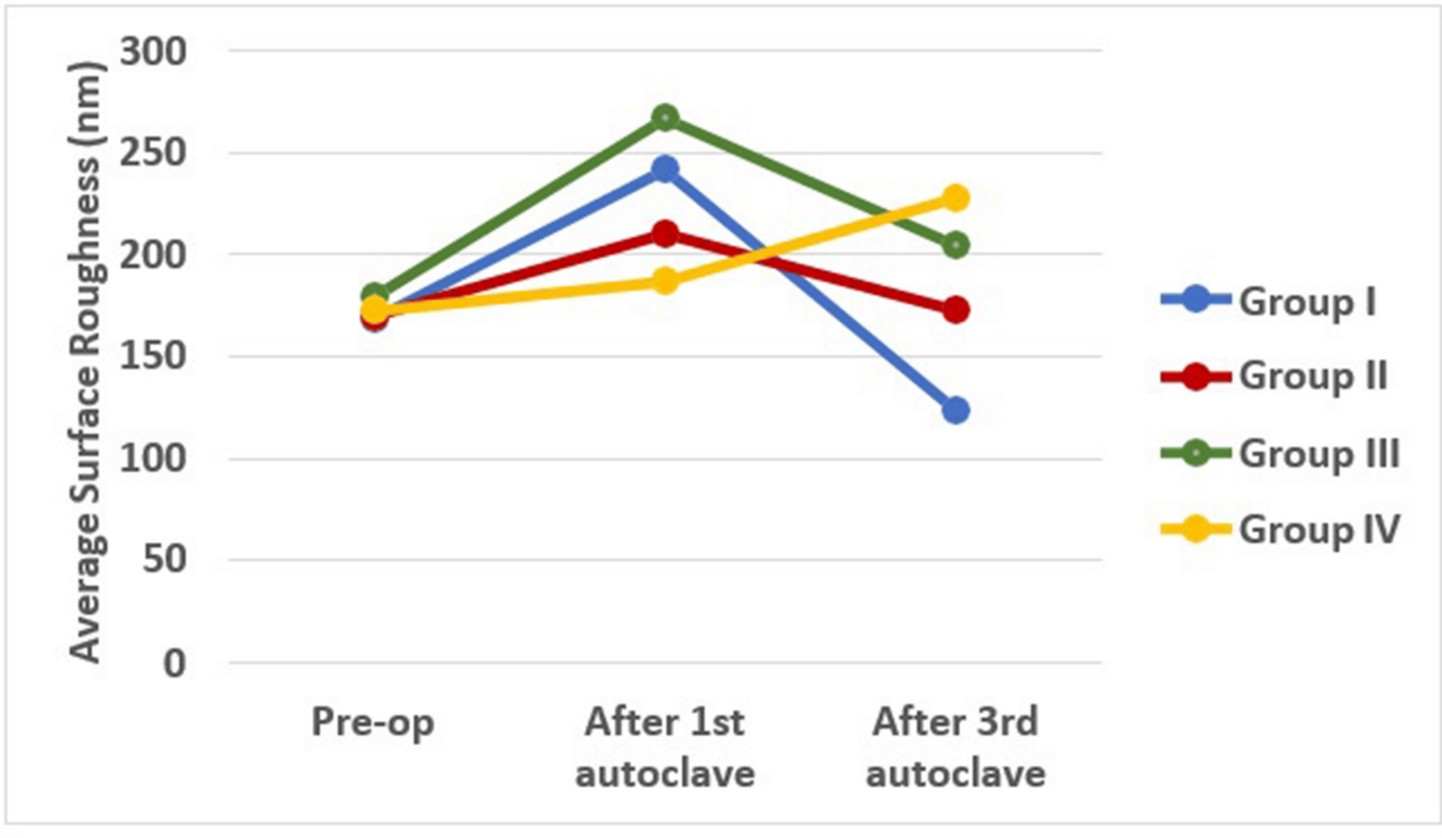
Average surface roughness between control and experimental groups in nanometers (nm) Group I: Hyflex EDM control group, Group II: WaveOne gold control group, Group III: Hyflex EDM experimental group, Group IV: WaveOne gold experimental group

**Figure 2:**
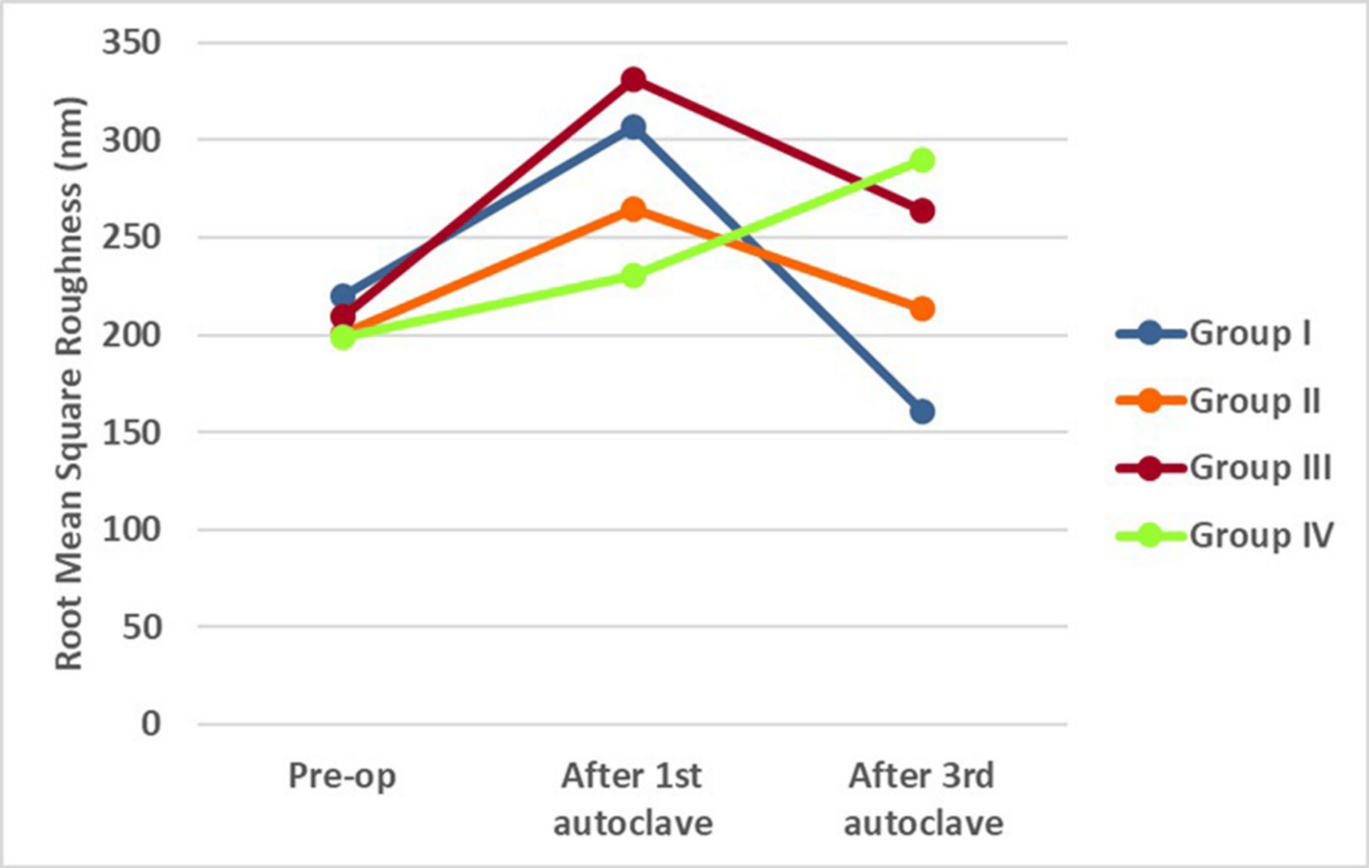
Root mean square roughness between control and experimental groups in nanometers (nm). Group I: Hyflex EDM control group, Group II: WaveOne gold  control  group, Group  III  : Hyflex EDM experimental group, Group IV : WaveOne gold  experimental  group

The intergroup comparison of average surface roughness and root mean square roughness was analyzed using One-Way Analysis of Variance (ANOVA). This is the method of choice when comparing the mean values of multiple groups in order to determine whether significant differences exist among them. The analysis revealed non-significant differences in both Ra and Rq values among the four groups (p<0.05) (Table 1, Figures 1, 2).

**Table 1:** Intergroup comparison of average and root mean square surface roughness Group I - Hyflex EDM control, Group II - WaveOne Gold control, Group III - Hyflex EDM experimental, Group IV - WaveOne Gold experimental NS – Statistically not significant (p>0.05).

			Avg. surface roughness (nm)		Root mean square roughness (nm)	
Cycles	Groups	n	One-way ANOVA test value	P-value	One-way ANOVA test value	P-value
Pre-op	I	6	0.015	0.997 [NS]	0.050	0.985 [NS]
	II	6				
	III	6				
	IV	6				
	Total	24				
After 1^st^ autoclave	I	6	0.634	0.602 [NS]	0.602	0.621 [NS]
	II	6				
	III	6				
	IV	6				
	Total	24				
After 3^rd^ autoclave	I	6	2.100	0.132 [NS]	2.305	0.108 [NS]
	II	6				
	III	6				
	IV	6				
	Total	24				

​​​Intragroup comparison

For intragroup comparison, the focus was on evaluating the changes in Ra and Rq within each group over the three time points: pre-op, after the first autoclave, and after the third autoclave. Repeated measures ANOVA was employed for this analysis.

**Figure 3:**
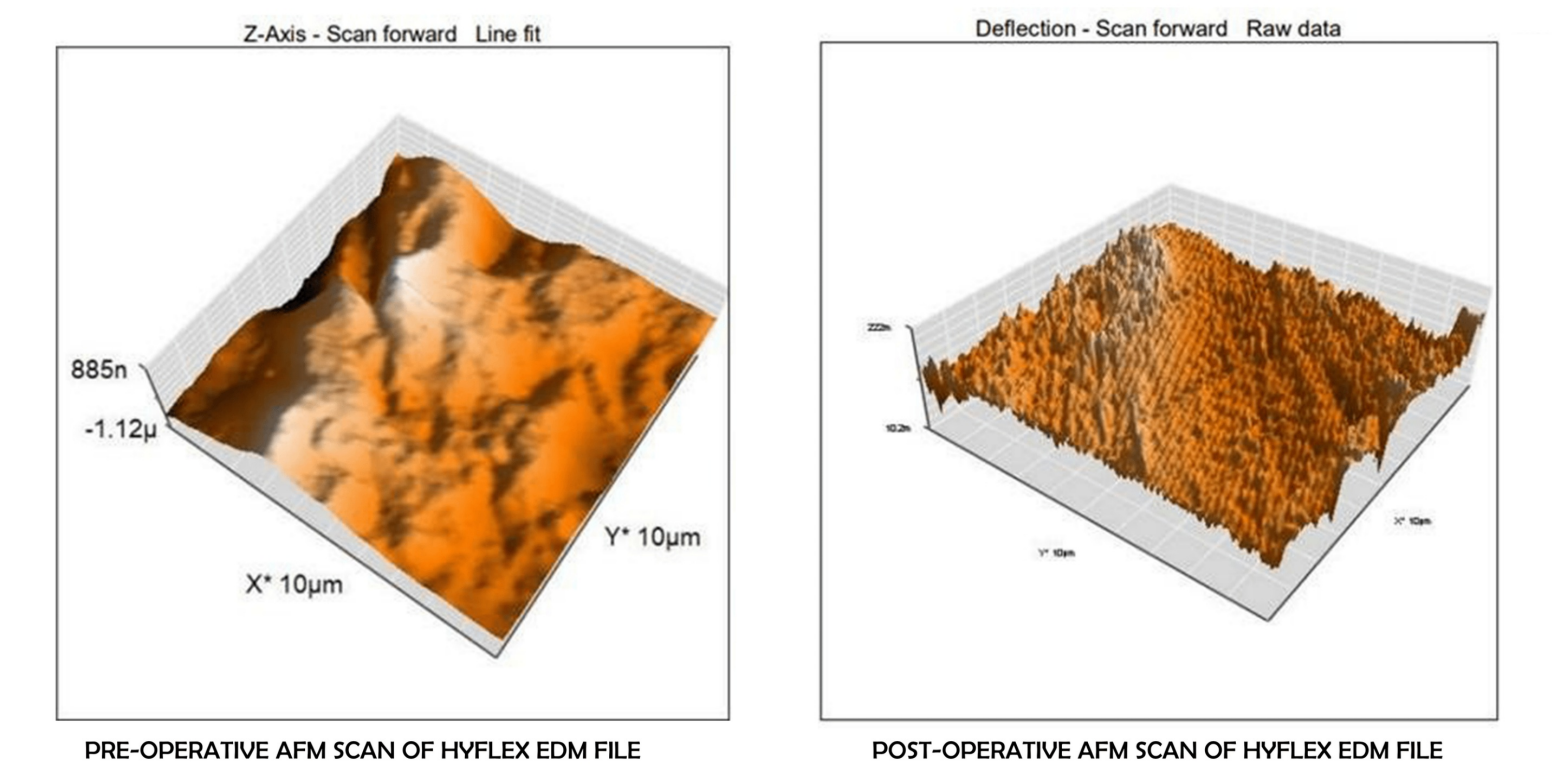
Pre-operative and post-operative surface roughness of Hyflex EDM files as seen under an atomic force microscope.

**Figure 4:**
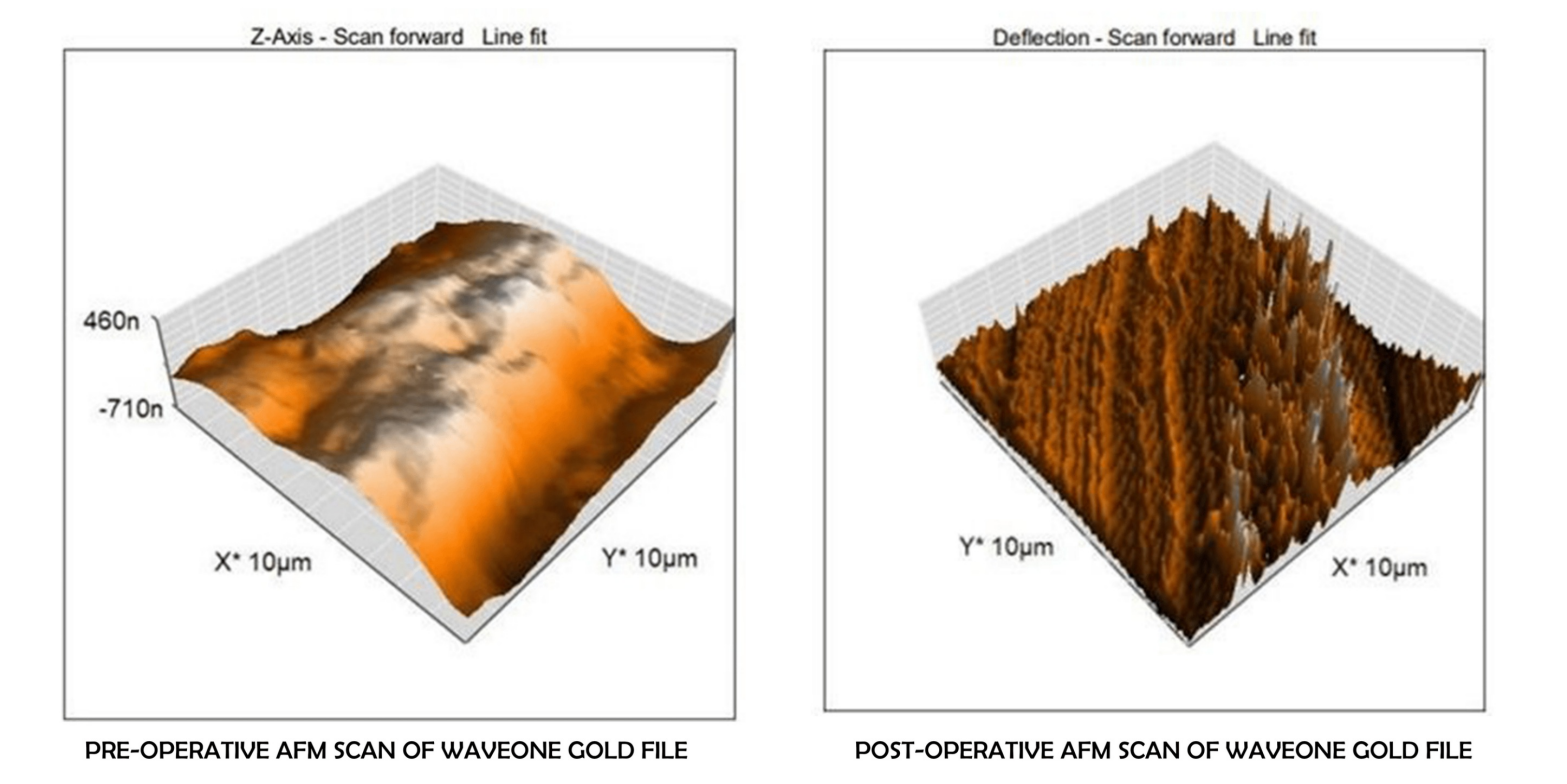
Pre-operative and post-operative surface roughness of WaveOne Gold files as seen under an atomic force microscope

The results of the repeated measures ANOVA indicated non-significant changes in surface roughness within each group across the different time points (p<0.05) (Table 2). To further explore these changes, “Tests of Within-Subjects Effects” and the “Tests of Within-Subjects Contrasts” were done. Both these tests showed that there is no significant difference between the means of average surface roughness and root mean square roughness at various time points; and similarly, between various time points in different groups (Sphericity assumed: p = 0.095; p = 0.516, respectively). The differences in the surface roughness of the files can be seen in the examples of the two file systems used in this study (Figures 3, 4). 

**Table 2:** Intragroup comparison of average and root mean square roughness NS – Statistically not significant (p>0.05)

		Average surface roughness (nm)		Root mean square roughness (nm)	
Groups	Time	Mean + SD	Repeated measures ANOVA test: P-value	Mean + SD	Repeated measures ANOVA test: P-value
Hyflex EDM control	Pre-op	168.36 ± 103.98	0.090 [NS]	219.87 ± 123.97	0.088 [NS]
	After 1^st^ autoclave	241.59 ± 106.19		306.59 ± 131.98	
	After 3^rd^ autoclave	123.56 ± 18.75		160.86 ± 30.37	
	Total	177.84 + 95.42		229.11 + 117.08	
WaveOne Gold control	Pre-op	169.85 ± 87.54	0.801 [NS]	200.08 ± 100.66	0.741 [NS]
	After 1^st^ autoclave	210.29 ± 150.96		264.41 ± 209.48	
	After 3^rd^ autoclave	172.65 ± 101.27		213.52 ± 117.05	
	Total	184.26 + 110.05		226.00 + 143.98	
Hyflex EDM experimental	Pre-op	179.24 ± 97.62	0.276 [NS]	209.58 ± 93.59	0.179 [NS]
	After 1^st^ autoclave	266.75 ± 105.84		331.10 ± 127.95	
	After 3^rd^ autoclave	204.53 ± 72.24		263.71 ± 96.77	
	Total	216.84 + 95.21		268.13 + 112.97	
WaveOne Gold experimental	Pre-op	172.33 ± 91.02	0.436 [NS]	198.98 ± 105.11	0.222 [NS]
	After 1^st^ autoclave	186.81 ± 35.47		230.20 ± 45.25	
	After 3^rd^ autoclave	227.86 ± 86.36		289.59 ± 98.81	
	Total	195.67 + 74.74		239.59 + 90.66	

## Discussion

The flexibility and great cutting efficiency of NiTi rotary files have led to considerable advancements in endodontic therapy [11]. Nevertheless, the surface topography of NiTi rotary files can be influenced by different variables. During the process of endodontic instrumentation, the application of mechanical friction can result in wear and the creation of surface irregularities [12-14], which leads to both a higher likelihood of file breakage and a decrease in cutting effectiveness [7].

Given the frequent reuse of equipment, it is imperative to sterilize them after each use to prevent cross-contamination among patients. However, there is ambiguity surrounding the possible alterations to the physical and mechanical characteristics of NiTi files when exposed to sterilization cycles using autoclaves or dry heat sterilizers [15]. Research has indicated a decline in the cutting effectiveness of NiTi instruments, potentially due to modifications in the instrument's surface.

According to a recent systematic review, the surface roughness of NiTi files is found to increase with autoclave sterilization. This could be due to the elevated oxygen levels on the surface of the file during multiple sterilization cycles, thereby increasing the surface roughness of the file [16]. This increase in roughness is found to correspond with the number of autoclaving cycles, resulting in increased cyclic fatigue, thereby decreasing the effectiveness of root canal treatment [17,18].

The assessment of surface roughness of NiTi rotary files offers valuable insights into surface defects, performance, and related constraints [19]. Consequently, researchers have investigated several methods, including SEM, AFM, and noncontact optical profilometry, to examine the surface structure of endodontic files [20-24].

AFM is a type of scanning probe microscopy that instantly creates a three-dimensional representation of the sample's surface on a computer screen and also saves the experimental quantitative data in digital format. AFM was used in this study because it is both sensitive and dependable, providing an appropriate method for obtaining data on the surface topography of rotary NiTi files. The surface roughness values were calculated by considering the average surface roughness and root mean square roughness values of the files [9]. Thus, the present study aimed to evaluate the difference between the effect of cyclic fatigue and autoclaving cycles on the surface properties of files using AFM.

In the present study, a pre-operative intergroup comparison was conducted to assess the mean values of average surface roughness and root mean square roughness, establishing baseline data. The average surface roughness in the preoperative stage was highest in Group III (Hyflex EDM experimental), followed by Group IV (WaveOne Gold experimental), Group II (WaveOne Gold control), and was least in Group I (Hyflex EDM control). However, the differences between the mean values of the four groups were statistically non-significant.

Similarly, for the mean values of root mean square surface roughness, the highest values were observed in Group I (Hyflex EDM control), followed by Group III (Hyflex EDM experimental), Group II (WaveOne Gold control), and the least in Group IV (WaveOne Gold experimental). Again, the differences in mean values were statistically non-significant. This indicates that at baseline, the files used in this study had similar surface characteristics.

In the current study, all files in Group I (Hyflex EDM control) and Group II (Wave One Gold control) were subjected to the first autoclave cycle. The mean values of average surface roughness and root mean square roughness of files in both groups showed an increase from the pre-operative values. This was consistent with a study by Yılmaz et al. that demonstrated an increase in surface roughness of files after a single autoclave cycle [25].

Files in Group III (Hyflex EDM experimental) and Group IV (WaveOne Gold experimental) were used in a simulated curved canal and then subjected to an autoclave cycle. The mean differences in surface and root mean square roughness values of both file groups showed an increase when compared to their baseline values. This finding aligned with previous studies that have shown an increase in surface roughness of Hyflex EDM (Group III) and WaveOne Gold (Group IV) after usage in curved canals.

The files from Group I (Hyflex EDM control) and Group II (Wave One Gold control) were then subjected to a second and third autoclave cycle. After the third autoclave cycle, the files were scanned using AFM. Both groups experienced a decrease in mean average surface roughness and root mean square roughness values compared to the first autoclave cycle. This was in contrast to previous studies, which showed an increase in surface roughness values. This discrepancy could be due to differences in autoclaving parameters or methods of testing. A study by Rapisarda et al. showed that repeated sterilizations using an autoclave contribute to changes in the cutting ability of the file [16].

Hyflex EDM experimental (Group III) files were used for a second time followed by autoclaving. After this, the files were used for a third time and autoclaved again. Following the third usage and autoclaving, the files were evaluated using AFM. The mean average surface roughness and root mean square roughness values decreased in relation to their initial usage values. This was in contrast to the study by Uslu et al. which showed increased surface roughness values for Hyflex EDM after instrumentation in curved canals [6].

WaveOne Gold experimental (Group IV) files were also used for a second time followed by autoclaving. After this, the files were used for a third time and autoclaved again. Following the third usage and autoclaving, the files were evaluated using AFM. The mean values of average surface roughness and root mean square roughness were observed to be higher. This was in agreement with studies by Zafar et al. and AlRahabi et al. that showed an increase in surface roughness values after instrumentation [26,27]. Furthermore, Özyürek et al. employed AFM to conduct a comparative analysis of the surface topography of WaveOne Gold (WOG) and WaveOne (WO). They observed that WOG exhibited greater surface porosity values compared to WO after undergoing instrumentation, which resulted in an increase in surface roughness of the WaveOne Gold files [28].

On comparing the files in Groups I (Hyflex EDM control) and III (Hyflex EDM experimental) after the third usage, an increase in surface roughness was observed following the third usage. Similarly, comparing the files in Groups II (WaveOne Gold control) and IV (WaveOne Gold experimental) revealed an increase in surface roughness after the third usage. This indicates that the increase in surface roughness directly correlates with usage in curved canals.

In both intragroup and intergroup comparisons, the differences in mean values showed no significant differences between the two differently manufactured file systems used in this study. The manufacturing procedures of these file systems are claimed to provide superior qualities compared to standard NiTi files, particularly in terms of enhanced strength and fatigue resistance for usage in curved canals. However, the results of this research show that neither of the file groups were superior to each other.

The present study had several limitations. Firstly, the study was conducted using simulated resin blocks with a curved canal configuration, rather than natural teeth. A study by Ferreira et al. observed groove and cutting-edge deformations, as well as increased values of surface roughness in files, after instrumentation in artificial canals [17]. This suggests that the findings in artificial canals may not fully replicate the conditions in natural teeth.

Secondly, the present study employed AFM to examine the surface properties of the files. While AFM provides detailed topographical information, it can only record a small section of the file at a time [29]. This limitation means that the AFM analysis may not represent the overall surface roughness of the entire file, potentially missing variations that occur along the length of the file.

## Conclusions

Based on the results of the present study, it can be concluded that there was no statistical significance achieved between the groups tested. Hyflex EDM and WaveOne Gold files which have different manufacturing processes and file motions, showed similar levels of surface changes when subjected to multiple usage and autoclaving cycles. Thus, the null hypothesis of this study is accepted.
